# Conflicting effects of atazanavir therapy on atherosclerotic risk factors in stable HIV patients: A randomized trial of regimen switch to atazanavir

**DOI:** 10.1371/journal.pone.0181993

**Published:** 2017-10-12

**Authors:** Joshua A. Beckman, Brian R. Wood, Kevin L. Ard, Christin N. Price, Daniel A. Solomon, Jonah P. Zuflacht, Jessica Milian, Joshua C. Prenner, Paul E. Sax

**Affiliations:** 1 Cardiovascular Division, Vanderbilt University Medical Center, Nashville, Tennessee, United States of America; 2 Division of Infectious Disease, Brigham and Women’s Hospital, Boston, Massachusetts, United States of America; 3 Cardiovascular Division, Brigham and Women’s Hospital, Boston, Massachusetts, United States of America; Azienda Ospedaliera Universitaria di Perugia, ITALY

## Abstract

Bilirubin acts as a potent endogenous antioxidant, with higher concentrations associated with lower rates of CVD; the antiretroviral drug atazanavir (ATV) increases bilirubin levels but may also increase von Willebrand factor levels. We tested the hypothesis that increasing endogenous bilirubin using ATV would improve cardiometabolic risk factors and vascular function in older patients with HIV. Ninety participants were enrolled in two study protocols. In protocol 1, we evaluated markers of inflammation, thrombosis, and conduit artery endothelial function in subjects on non-ATV containing regimens. Participants were randomly assigned to continue baseline treatment or switch to an ATV-based regimen. Measurements were made at baseline and 28 days. In the protocol 2, we enrolled 30 subjects who received atazanavir for more than one year and were compared to the aim 1 protocol subjects at baseline. 60 subjects were enrolled in the first protocol (mean age 53, +/- 6 years), with 31 randomized to ATV and 29 continuing baseline treatment. Atazanavir significantly increased serum total bilirubin levels (p<0.001) and acutely but not chronically plasma total antioxidant capacity (p<0.001). An increase in von Willebrand Factor (p<0.001) and reduction in hs-CRP (p = 0.034) were noted. No changes were seen in either flow-mediated endothelium-dependent or vasodilation. In cross-sectional analysis (second protocol), similar findings were seen in the baseline attributes of non-atazanavir-based and long-term atazanavir users. Increasing serum bilirubin levels with atazanavir in subjects with HIV reduces hs-CRP, temporarily reduces oxidative stress, but increases von Willebrand Factor. Atazanavir does not improve endothelial function of conduit arteries.

**Trial registration:** ClinicalTrials.gov NCT03019783.

## Introduction

Effective antiretroviral therapy (ART) has dramatically reduced AIDS-related morbidity and mortality for those with HIV [1]. With increased survival, HIV-infected patients are at increased risk for diseases of aging, including cardiovascular disease. Some studies suggest that cardiovascular disease is more common in this population than in HIV-negative age-matched controls, with possible contributors including residual excess inflammation and immune activation despite effective ART. Additional potential factors include dyslipidemia, certain antiretroviral agents, and a high prevalence of modifiable CV risk factors, in particular smoking [2–9].

While some early HIV protease inhibitors (PIs) unfavorably influenced cardiovascular risk due to deleterious effects on lipids and insulin resistance, this has not been observed with the PI atazanavir [10]. Atazanavir raises unconjugated bilirubin—a potent intracellular antioxidant—through inhibition of the enzyme uridine diphosphate glucuronyltransferase (UGT) 1A1. We have demonstrated that higher levels of plasma bilirubin, within the normal range, are associated with reduced rates of stroke and peripheral artery disease in the general population [11, 12]. In addition, patients with Gilbert Syndrome (chronic elevation of bilirubin as a result of genetically reduced UGT1A1) have a lower rate of myocardial infarction compared with age-matched controls [13].

However, recent experimental data suggests that bilirubin inhibits A Disintegrin-like and Metalloprotease with Thrombospondin type-1 motifs (ADAMTS)-13 and may raise von Willebrand factor levels [14] offsetting this cardiovascular benefit. Based on these observations, we hypothesized that the use of atazanavir in an older stable HIV population would reduce oxidative stress and increase von Willebrand factor levels rendering an unclear impact on vascular function compared with continuing current therapy. We compared the oxidative stress, von Willebrand factors, and vascular function subjects randomly assigned to continue their current ART regimen or switch to a regimen containing atazanavir. We also conducted a cross-sectional analysis comparing these outcomes at baseline (prior to randomization) to a separate group of subjects receiving long-term (≥ 1 year) atazanavir therapy.

## Materials and methods

### Subject selection

Subjects were recruited from affiliated practices and local advertisements. For the randomized study, inclusion criteria included age ≥ 45 years, stable non-atazanavir-containing regimen consisting of co-formulated tenofovir disproxil fumarate/emtricitabine (TDF/FTC) as the nucleoside reverse transcriptase inhibitor (NRTI)s plus a third active agent for 3 months or longer. The third agent could be any FDA-approved protease inhibitor (PI), non-nucleoside reverse transcriptase inhibitor (NNRTI), or integrase strand transfer inhibitor (INSTI). Patients were required to have an HIV RNA < 50 cop/mL at screening and at least once during the prior year, and no treatment interruptions > 7 days in the 3 months prior to study entry. For the cross-sectional study, subjects taking atazanavir-based therapy for at least one year were recruited. Subjects were excluded if they had prior treatment failure on or intolerance to atazanavir, known or suspected resistance to atazanavir, evidence of unstable cardiovascular disease within 1 year, renal or liver disease, a history Gilbert Syndrome or elevated bilirubin levels (> 1.5 mg/dL) at baseline, current receipt of ART different from co-formulated TDF/FTC plus a third agent (PI, NNRTI, or INSTI) regimen, current receipt of proton-pump inhibitor therapy, or recent initiation of hormones or immunomodulators. Active drug abuse, smoking, pregnancy, or use of medications that interfere with atazanavir precluded participation. All subjects provided written informed consent. The protocol was approved by the Partners Human Research Committee of Brigham and Women’s Hospital, all subjects provided informed consent, and all study visits occurred at Brigham and Women’s Hospital. This study was approved by the Human Research Committee on November 21, 2011, patient recruitment began in December 2011, and was completed in May 2015. This study was registered when Section 801 of the Food and Drug Administration Amendments Act changed the description of clinical trials in September 2016 after patient recruitment began. Prior to this, we were not required by the Institutional Review Board to register physiological studies.

### Protocols

Protocol 1: In this parallel-design trial, 60 subjects taking non-atazanavir-based regimens were randomly allocated by the investigational drug pharmacy to remain on their original treatment or substitute atazanavir (Reyataz, Bristol Myers Squibb, Plainsboro, NJ) and ritonavir for the non-TDF/FTC component of the regimen; TDF/FTC was continued for all patients (ritonavir acts solely as a pharmacokinetic booster in this context). Subjects were randomly allocated to treatment arms in a 1:1 ratio based on a block size of 4 by the Brigham and Women’s Hospital Investigational Pharmacy. The treatment was applied by an HIV specialist (PS), but blinded to the cardiovascular specialist (JB). The subjects who were switched received atazanavir 300 mg with ritonavir 100 mg daily for 28 days. After the 28 days, HIV therapy was continued as per the subject’s treating physician. Subjects underwent analysis for markers of lipids, oxidative stress, inflammation, thrombosis, and vascular function occurred on days 1 and 28.

Protocol 2: In this cross-sectional analysis, the parameters of interest were compared in 30 subjects who received atazanavir for more than a year to the baseline attributes of the 60 subjects on non-atazanavir-based regimens recruited for protocol 1.

### Laboratory analyses

Blood was collected into Vacutainer tubes (Becton Dickinson) for chemistry and hematologic analyses at Brigham and Women’s Hospital Clinical Laboratories. Plasma samples were stored for subsequent analysis at -80°C from Vacutainer tubes containing K2 EDTA 7.2 mg/4 mL whole blood following centrifugation (1,200 g) at 4°C for 10 minutes. Plasma antioxidant capacity was assessed by ferric reducing ability of plasma (FRAP) assay at the Clinical & Epidemiologic Research Laboratory of Boston Children’s Hospital [15].

ADAMTS13 (A Disintegrin-like and Metalloprotease with Thrombospondin type-1 motifs 13) was measured with a fluorometric method from AnaSpec Inc (Freemont, CA). The assay employs an internally quenched vWF73 FRET peptide substrate for the detection of enzyme activity. High Sensitivity C-Reactive Protein (hs-CRP) was determined using an immunoturbidimetric assay on the Roche P Modular system (Roche Diagnostics—Indianapolis, IN), using reagents and calibrators from Roche. The FRAP kit (Ferric Reducing Ability of Plasma) from Arbor Assays (Ann Arbor, MI) is a colorimetric assay used to measure the antioxidant status of plasma samples. Interleukin-6 (IL-6) was measured by an ultra-sensitive ELISA assay from R & D Systems, Minneapolis, MN. Myeloperoxidase (MPO) was measured by an ELISA assay from Alpco Diagnostics (Salem, NH). Soluble intercellular adhesion molecule-1 (sICAM-1) was measured by an ELISA assay (R & D Systems, Minneapolis, MN). Soluble vascular cell adhesion molecule-1 (sVCAM-1) was measured by an ELISA assay (R & D Systems, Minneapolis, MN). TNFα-receptor II was measured by an ELISA assay from R & D Systems. Von Willebrand factor (vWF) antigen was measured by an ELISA assay from American Diagnostica (American Diagnostica—Greenwich, CT). The MPO, sICAM-1, sVCAM-1, TNFα-receptor II, and vWF assays employ the quantitative sandwich enzyme immunoassay technique.

The homeostatic model assessment-estimated insulin resistance (HOMA_IR_) was calculated using the following equation: fasting glucose (mg/dL) x fasting insulin (mU/mL) / 405.

### Vascular reactivity testing

Subjects were studied in the morning after overnight fast. All vascular studies were performed in a quiet, temperature-controlled, dimly lit room after the subject rested supine for a minimum of 5 minutes, using an upper-arm sphygmomanometric cuff position, as we have previously performed and according to guidelines [16–19]. High-resolution B-mode ultrasonography using a 7.5 MHz linear array probe (Vivid 7, General Electric) was used to image the brachial artery. Images were obtained using an electrocardiographic R-wave trigger for end diastole. Reactive hyperemia was induced through five minutes of cuff suprasystolic pressure inflation. Flow-mediated, endothelium-dependent vasodilation was assessed 60 to 70 seconds after cuff deflation. We have shown that vasodilation at this time point is largely dependent upon endothelium-derived nitric oxide [20, 21]. Vascular function analyses were performed blinded to study visit.

Ten minutes after cuff release endothelium-independent vasodilation was assessed. The brachial artery was imaged before and 3 minutes after sublingual administration of 0.4 mg of nitroglycerin (Nitrostat, Parke-David, New York, NY). Brachial artery blood flow velocity was determined via pulsed Doppler velocity-time integral measurement at all time points. Nitroglycerin was not administered if the subject’s systolic blood pressure was < 100 mmHg or heart rate was < 50 beats/min. Analysis was performed using Information Integrity custom-made image acquisition and analysis software (Information Integrity, Stow, MA).

### Statistical methods

The effect and sample sizes were modeled based on previous work with another antioxidant [22]. Descriptive measures are reported as mean ± standard deviation (SD). Vascular function parameters were compared by analysis of variance in Protocol 1 and paired t-test in Protocol 2. Baseline characteristics are described as mean and SD. Comparison of baseline characteristics between groups was performed using ANOVA, Kruskal–Wallis equality-of-populations test, or χ^2^ test, as appropriate. The primary outcome assessment was brachial artery flow-mediated vasodilation (FMD) response during atazanavir-based therapy compared with non-atazanavir-based-therapy. The distribution of FMD at both time points was normally distributed (p = NS by Shapiro–Wilk test); consequently, ANOVA was performed to assess the difference in mean FMD between atazanavir and non-atazanavir regimens. Other subgroup descriptive analyses were performed by paired Student t test or the Wilcoxon signed-rank test, as needed. Statistical significance was accepted at the 95% confidence level (P < 0.05. SPSS version 24 for MAC (IBM, Armonk, NY) was used for all analyses.

## Results

### Study participants

**Fig 1:**
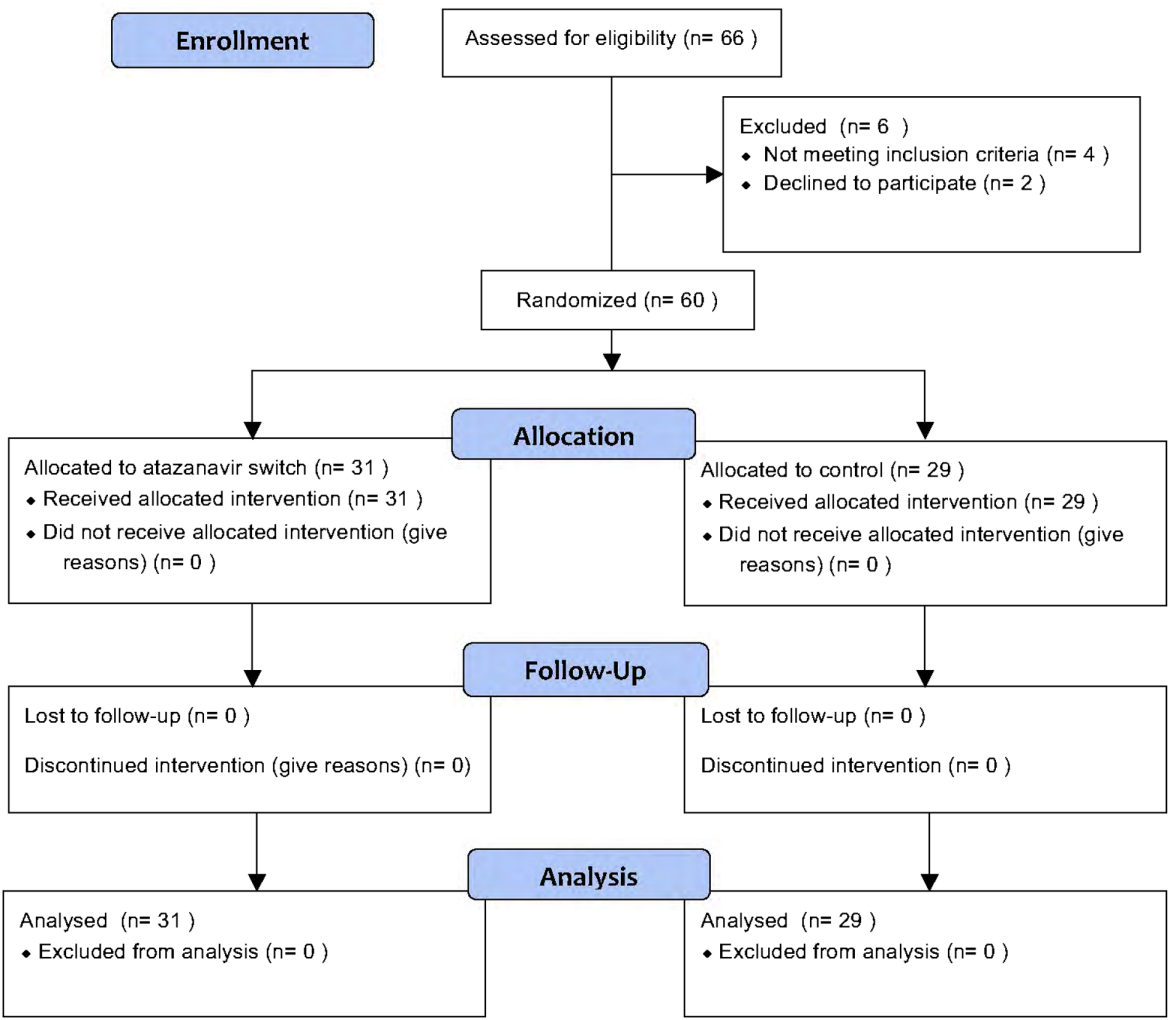
Consort flow diagram for treatment trial.

102 subjects with HIV signed consent. The first subject started in December 2011 and the last subject completed participation in May 2015. Nine did not meet study criteria after laboratory analysis and 3 did not show up for study visits (Fig 1). A total of 90 subjects completed the study, including 60 in Protocol 1 and 30 in Protocol 2. The study was considered completed when the full cohort had completed the protocol. Participants had a mean age of 53 years, included 18 women, and had normal blood pressure, lipid levels and renal function. The mean body mass index (BMI) was 27 kg/m^2^ and plasma HIV-1 RNA was 30 copies / ml in the atazanavir-naïve participants and 37 copies / ml in the atazanavir-treated participants. Between the two groups, there was no difference in insulin, glucose, or HOMA-IR, lipid levels, and ICAM-1 or IL-6.

### Treatment trial (Protocol 1)

In the parallel-design, randomized, blinded trial (Protocol 1), there was neither a significant change within group or between groups in renal function, lipids, insulin, glucose, HOMA-IR, complete blood counts, and blood pressure with a change to atazanavir compared with remaining on the original regimen. Treatment with atazanavir significantly increased total bilirubin by 1.6 ± 0.8 mg/dL compared to 0.0 ± 0.1 mg/dL for subjects who remained on their original regimen, p < 0.001 (Table 1). Allocation to atazanavir was associated with a reduction in hs-CRP and an increase in von Willebrand factor (vWF) and FRAP when compared to treatment with the baseline regimen. Increases in bilirubin were directly correlated with increases in FRAP (Pearson 0.397, p = 0.002) (Table 2).

**Table 1:** Baseline demographics, laboratories and vascular function parallel design trial (Protocol 1).

Parameter	No Regimen Switch SwitChange (n = 32)	Atazanavir Switch (n = 28)	p value
**N**	31	29	
**Age (y)**	53±5	53±7	0.36
**Sex (% Female)**	43%	21%	0.07
**BMI (kg/m^2^)**	27±6	28±6	0.55
**Race (% Caucasian)**	42%	60%	0.26
**Hispanic (%)**	13%	13%	0.84
**Systolic Blood Pressure (mm Hg)**	125±15	125±17	0.99
**Diastolic Blood Pressure (mm Hg)**	77±9	77±8	0.83
**Mean Arterial Pressure (mm Hg)**	92±10	91±10	0.70
**Total Cholesterol (mg/dl)**	182±30	182±35	0.97
**LDL (mg/dl)**	103±30	107±33	0.59
**HDL (mg/dl)**	53±19	50±17	0.59
**Triglycerides (mg/dl)**	141±73	135±68	0.84
**Plasma HIV-1 RNA (copies / ml)**	28±25	33±30	0.49
**Alkaline Phosphatase (IU/L)**	86±30	96±37	0.24
**AST (IU/L)**	29±14	28±13	0.89
**ALT (IU/L)**	31±13	36±27	0.35
**GGT (IU/L)**	44±31	93±162	0.10
**Total Bilirubin (mg/dl)**	0.3±0.2	0.4±0.2	0.35
**Direct Bilirubin (mg/dl)**	0.1±0.0	0.1±0.1	0.28
**Total Protein (g/dl)**	7.7±0.7	7.5±0.4	0.20
**Albumin (g/L)**	4.3±0.4	4.3±0.3	0.90
**LDH (IU/L)**	172±37	175±46	0.78
**CPK (IU/L)**	179±128	131±56	0.08
**BUN (mg/dl)**	14.1±3.9	14.7±3.8	0.56
**Creatinine (mg/dl)**	0.9±0.1	0.9±0.2	0.22
**eGFR (ml/min)**	111±24	106±29	0.31
**Glucose (mg/dL)**	95±19	88±20	0.17
**Insulin (IU/L)**	15±12	15±11	0.90
**HOMA-IR**	3.9±4.1	3.4±2.7	0.55
**WBC (1000 cells/mcl)**	5.8±1.8	6.3±2.2	0.40
**HGB (g/L)**	13.±1.2	14.2±1.2	0.009
**Platelets**	219±55	231±65	0.42
**Vascular Function**			
**Baseline Diameter (mm)**	3.7±0.6	3.6±0.7	0.58
**Reactive Hyperemia (FC)**	6.9±3.9	5.6±2.7	0.16
**Flow-Mediated Vasodilation (%)**	9.4±5.6	9.8±4.6	0.78
**Nitroglycerin-Mediated Vasodilation (%)**	19.1±7.4	15.6±6.8	0.11
**hs-CRP (mg/L)**	2.6±2.9	3.7±4.1	0.20
**VCAM (ng/mL)**	712±235	164±322	0.47
**ICAM-1 (ng/mL)**	239±100	277±103	0.16
**IL-6 (pg/mL)**	2.2±2.2	2.8±2.7	0.32
**Myeloperoxidase (ng/mL)**	20.4±16.6	30.5±34.9	0.15
**FRAP (uM)**	1142±254	1173±277	0.65
**ADAMTS-13 (RFU)**	0.16±0.03	0.17±0.04	0.40
**von Willebrand Factor (mU/mL)**	889±687	964±979	0.73
**Medications**			
**ARB or ACE I**	4	4	0.89
**Statin**	7	8	0.56
**Metformin**	4	1	0.22
**PPAR alpha agonist (%)**	2	2	0.89

**Table 2:** Change in expression for selected atherosclerotic markers in the parallel design trial.

Parameter	No Change in Therapy	Atazanavir	p
**Total Bilirubin (mg/dL)**	0.0±0.1	1.6±0.8	<0.001
**hs-CRP (mg/L)**	0.84±3.05	-0.53±4.6	0.034
**HOMA-IR**	-0.43±4.3	0.55±2.3	0.29
**HDL (mg/dL)**	-1.0±8.3	-4.5±10.8	0.16
**TRIGS (mg/dL)**	-6.9±52.9	19.1±80.2	0.14
**MPO** (ng/mL)	4.7±20.6	2.1±45.9	0.8
**IL-6** (pg/mL)	0.45±3.32	-0.11±3.28	0.5
**FRAP** (uM)	-21±271	219±213	<0.001
**VWF** (mU/mL)	-140±636	171±1026	0.05

Basal brachial artery diameter did not vary significantly between the atazanavir-treated and non-atazanavir treated groups (3.7 ± 0.7 mm at baseline and 3.9 ± 0.6 mm post-treatment, p = 0.64) (Table 3). There was no significant difference in flow-mediated, endothelium-dependent vasodilation between the two groups (9.6 ± 5.1% at baseline and 9.4 ± 5.5% post-treatment; p = 0.92). Nitroglycerin-mediated, endothelium-independent vasodilation was without significant difference between the groups. There was no significant difference in baseline diameter, reactive hyperemic stimulus, flow-mediated vasodilation, and nitroglycerin-mediated vasodilation at any time point, both within and between groups.

**Table 3:** Vascular function in the parallel design trial.

	No Change in Therapy		Atazanavir	
Parameter	Day 1	Day 28	Day 1	Day 28
**Baseline Diameter (mm)**	3.7 ± 0.6	3.7 ± 0.5	3.6 ± 0.7	3.6 ± 0.7
**Reactive Hyperemic Stimulus (FC)**	7.1 ± 3.9	5.9 ± 2.2	5.5 ± 2.8	4.8 ± 2.0
**Reactive Hyperemic Diameter (mm)**	4.0 ± 0.6	4.1 ± 0.6	3.9 ± .07	4.0 ± 0.7
**FMD (%)**	9.4 ± 5.6	9.6 ± 4.3	9.8 ± 4.6	10.6 ± 4.1
**NMD (%)**	18.6 ± 6.8	18.7 ± 6.1	16.0 ± 7.2	15.0 ± 5.4

### Cross-sectional analysis (Protocol 2)

Subjects receiving atazanavir for more than a year had increased total bilirubin levels compared with those never taking atazanavir (2.0 ±1.0 vs. 0.4 ± 0.2 mg / dL, p < 0.001) (Table 4). Direct bilirubin was modestly higher in the atazanavir group, as well, compared to those not taking atazanavir (0.2 ± 0.1 vs. 0.1 ± 0.1 mg / dL, p < 0.001). Despite the increase in bilirubin, there was no difference in oxidative stress, as measured by FRAP or myeloperoxidase levels. FRAP, 28 days after switch to atazanavir in the cross-sectional trial was greater than in the subjects taking it for more than a year (1393 ± 321 vs. 1103 ± 243, respectively (p = 0.037). The inflammatory profile was mixed, with lower levels of hs-CRP in subjects chronically taking atazanavir but higher levels of VCAM. Subjects taking atazanavir had higher levels of vWF compared to subjects not taking atazanavir and no difference in ADAMTS-13 levels. There was a significant correlation between total bilirubin and vWF levels (Pearson = 0.33, p = 0.001). There was no significant difference in baseline diameter, reactive hyperemic stimulus, flow-mediated vasodilation, and nitroglycerin-mediated vasodilation between groups.

**Table 4:** Cross-sectional comparisons (Protocol 2).

Parameter	Atazanavir Naïve (n = 60)	Atazanavir User (n = 30)	p value
**Age (y)**	53±6	53±6	0.63
**Sex (% Female)**	13%	33%	0.04
**BMI (kg/m^2^)**	27±6	28±6	0.70
**Race (% Caucasian)**	42%	60%	0.54
**Hispanic (%)**	13%	13%	1.0
**Systolic Blood Pressure (mm Hg)**	125±16	126±16	0.75
**Diastolic Blood Pressure (mm Hg)**	77±8	79±11	0.2
**Mean Arterial Pressure (mm Hg)**	91±10	93±14	0.41
**Total Cholesterol (mg/dl)**	182±32	172±33	0.19
**LDL (mg/dl)**	105±31	97±31	0.28
**HDL (mg/dl)**	52±18	49±23	0.60
**Triglycerides (mg/dl)**	141±73	135±68	0.68
**Plasma HIV-1 RNA (copies / ml)**	30±27	37±25	0.27
**Alkaline Phosphatase (IU/L)**	91±34	91±19	0.94
**AST (IU/L)**	29±13	28±13	0.96
**ALT (IU/L)**	33±21	33±25	0.97
**GGT (IU/L)**	67±114	39±38	0.20
**Total Bilirubin (mg/dl)**	0.4±0.2	2.0±1.0	<0.001
**Direct Bilirubin (mg/dl)**	0.1±0.1	0.2±0.1	<0.001
**Total Protein (g/dl)**	7.6±0.6	7.4±0.5	0.037
**Albumin (g/L)**	4.3±0.3	4.2±0.2	0.90
**LDH (IU/L)**	173±41	176±43	0.74
**CPK (IU/L)**	157±103	173±113	0.50
**BUN (mg/dl)**	14.4±3.8	15.9±5.4	0.14
**Creatinine (mg/dl)**	0.9±0.2	1.0±0.2	0.01
**eGFR (ml/min)**	109±26	107±28	0.71
**Glucose (mg/dL)**	92±20	94±33	0.65
**Insulin (IU/L)**	15±11	12±7	0.16
**HOMA-IR**	3.7±3.5	3.0±2.6	0.38
**WBC (1000 cells/mcl)**	6.0±2.0	6.3±1.9	0.55
**HGB (g/L)**	15.9±16.7	14.4±12.1	0.01
**Platelets**	225±60	207±54	0.16
**Vascular Function**			
**Baseline Diameter (mm)**	3.7±0.7	3.9±0.6	0.07
**Reactive Hyperemia (FC)**	6.3±3.5	6.1±2.9	0.86
**Flow-Mediated Vasodilation (%)**	9.6±5.1	9.4±5.5	0.18
**Nitroglycerin-Mediated Vasodilation (%)**	17.4±7.3	17.1±11.7	0.92
**hs-CRP (mg/L)**	3.1±3.5	1.9±2.0	0.05
**VCAM (ng/mL)**	736±278	870±297	0.04
**ICAM-1 (ng/mL)**	257±102	277±108	0.39
**IL-6 (pg/mL)**	2.5±2.4	2.3±2.2	0.68
**Myeloperoxidase (ng/mL)**	25.1±27.0	32.6±39.0	0.30
**FRAP (uM)**	1157±263	1103±243	0.35
**ADAMTS-13 (RFU)**	0.17 ± 0.03	0.17 ± .0.4	0.68
**von Willebrand Factor (mU/mL)**	924±830	1591±922	0.001
**Medications**			
**ARB or ACE I**	8	6	0.72
**Statin**	15	5	0.38
**Metformin**	5	4	0.46
**PPAR alpha agonist (%)**	4	0	0.15

## Discussion

Through inhibition of UGT1A1, atazanavir induces increases in bilirubin, occasionally significant enough to merit discontinuation secondary to jaundice [23–25]. However, the increase in bilirubin could also have beneficial effects, as bilirubin is a well-described potent endogenous antioxidant [26, 27]. Atazanavir, presumably through its increase in bilirubin, could improve the systemic atherosclerotic environment through improvements in lipid profile, inflammation, and oxidative stress[25, 28–30]. In support of this hypothesis, treatment with atazanavir slowed-progression in carotid intima media thickness compared to treatments with darunavir (another protease inhibitor] and raltegravir (an integrase inhibitor)[31, 32]. In the D:A:D study of more than 49,000 HIV-positive patients, treatment with atazanavir did not increase the risk of myocardial infarction or stroke, suggesting a safer cardiovascular profile for atazanavir compared with other HIV protease inhibitors [10].

We and others have reported an association between high-normal levels of unconjugated bilirubin and decreased rates of atherosclerosis in the general population. Moreover, acute increases in bilirubin have been associated with improvements in oxidative stress and endothelial function in patients who have type 2 diabetes. However, our current work raises questions about the value of pharmacologic-induced increases in bilirubin. This investigation demonstrates that while atazanavir-based increases in bilirubin exerted short-term improvements in oxidative stress and a modest short-term impact on inflammation, these benefits were not in evidence long-term. In addition, bilirubin-mediated inhibition of ADAMTS-13 [14] increased vWF levels.

The impact of increased bilirubin on vWF levels is a novel finding in humans in vivo. ADAMTS-13 cleaves vWF at the Tyrosine 1605-Methionine 1606 bond. When this cleavage occurs in excess, as in von Willebrand disease or aortic valvular stenosis, a bleeding diathesis ensues [33, 34]. When cleavage is inhibited, resulting in increased vWF, thrombotic thrombocytopenic purpura may occur [35–37]. Unconjugated bilirubin has been shown in vitro to inhibit vWF cleavage by ADAMTS-13 [14]. Our data are the first to support this finding in humans. We found higher levels of vWF after 28 days of treatment and even higher levels in the long-term cross-sectional analysis in patients treated with atazanavir compared to those not on atazanavir. Further, there was a strong direct correlation in total bilirubin and vWF levels in the cross-sectional aim. The clinical significance of this finding is unclear. Our study breaks the link reported previously between vWF and endothelial function in the determination of endothelial health [38]. In addition, higher levels of vWF have been associated epidemiologically with atherosclerosis, in the coronary, cerebrovascular, and peripheral arterial beds [39–41]. The mean levels in our long-term atazanavir-treated subjects are similar to the levels in the patients with cardiovascular events in these studies. Thus, the net effect of pharmacologically-mediated bilirubin raising has yet to be fully elucidated.

The effect of atazanavir on oxidative stress is complex. In our current work, the switch to an atazanavir-based regimen was associated with an increase in the ferric reducing ability of plasma (less oxidative stress) at 4 weeks, but there was no variation in the cross-sectional comparison of subjects taking atazanavir-based regimens versus other regimens for at least a year. Similarly, Flammer and colleagues reported no difference in oxidative stress 24 weeks after switching to atazanavir [42]. Our findings stand in contrast to studies in subjects with Gilbert syndrome who have decreased levels of oxidative stress as measured by advanced glycation end-products, urinary excretion of biopyrrins, reduced concentrations of oxidized LDL, and serum concentrations of malondialdehyde-modified LDL [43–46]. The variation in oxidative stress may reside in additional targets of atazanavir. Recent data suggests that protease inhibitors increase mitochondrial superoxide anion generation that may offset, in the vascular endothelium, any beneficial effect of higher circulating bilirubin [47, 48]. The effect of atazanavir on the mitochondria would also provide an explanation for an early decrease in oxidative stress through UGT1A1 only to be eliminated by later induction of mitochondrial superoxide anion production. This provides one explanation for the variance between the liver-specific Gilbert syndrome-mediated increases in bilirubin and target/off-target effects of medications that increase bilirubin.

### Atazanavir and endothelial function

Our data in older subjects with well-treated HIV extends previous reports in younger cohorts demonstrating no improvement in endothelial function [42, 49]. Moreover, our data verify a lack of benefit on endothelial function associated with long-term therapy in older subjects [50]. The results confirm our prior experience with atazanavir in patients with type 1 diabetes [28]. The lack of change in endothelial function is surprising given that increases in bilirubin decreased oxidative stress. The lack of effect may occur as a result of the mechanism by which bilirubin was increased. Heme Oxygenases (HO), both the inducible, HO-1, and constitutively expressed versions, HO-2, degrade heme to biliverdin. Biliverdin is, in turn, rapidly converted by biliverdin reductase to bilirubin. Bilirubin is then conjugated in the liver by UGT1A1 to a water soluble form destined for bodily elimination [51]. There are two well-described mechanisms for increasing bilirubin: inducing HO-1 and inhibiting UGT1A1. As HO-1 is expressed in endothelial cells, its induction would be predicted to increase bilirubin production. Both curcumin and peroxisome proliferator-activated receptor (PPAR)-alpha agonists induce HO-1 and have been shown in animals models to improve endothelial function [52–55] and improve vascular function in patients with diabetes [56, 57]. In contrast, a strategy of increasing bilirubin by blocking its conjugation in the liver has failed multiple times to improve endothelial function in subjects with HIV [30, 42, 49, 58] and type 1 diabetes [28], but had its single success in a 4-day study of subjects with type 2 diabetes [59]. It is possible that the success of the last study occurred because of factors particular to type 2 diabetes (its microvessel rather than conduit artery focus), the duration of the study (which was shorter than the length of time necessary to increase mitochondrial superoxide production), or because of random chance. Our data concur with most investigations in the finding that atazanavir does not improve conduit artery flow-mediated, endothelium-dependent vasodilation compared with other HIV treatment regimens.

## Conclusions

We demonstrate that atazanavir-based treatment of HIV temporarily improves oxidative stress and inflammation, but increases vWF levels, and has no effect on vascular function or the bioavailability of endothelium-derived nitric oxide. These effects suggest a complex pharmacological impact of atazanavir and raises questions about whether atazanavir treatment would reduce cardiovascular endpoints in HIV patients.

## Supporting information

S1 File CONSORT 2010 checklist. This is the CONSORT checklist.
(DOC)

S2 File Research proposal. This is the research proposal.
(DOCX)

S3 File Dataset. This is a limited dataset from the study.
(XLSX)
