# Supplementary material for: Conflicting effects of atazanavir therapy on atherosclerotic risk factors in stable HIV patients: A randomized trial of regimen switch to atazanavir
Source: PLoS One. 2017 Oct 12;12(10):e0181993. doi: 10.1371/journal.pone.0181993 (PMC5638209; doi:10.1371/journal.pone.0181993)
Supplement: S2 File — This is the research proposal. (DOCX) [file pone.0181993.s002.docx]

**Study title**: Atazanavir and Endothelial Function in Older HIV Patients

**Investigator names**: Joshua A. Beckman, MD and Paul E. Sax, MD

**Institution**: Brigham and Women’s Hospital

**Support type**: Drug and funds.

**Study rationale**

Human immunodeficiency virus (HIV)-related mortality has dropped significantly due to effective antiretroviral therapy. Epidemiological data suggest a less than 5% ten-year mortality for patients treated with anti-retroviral therapy (ART) (1). As a result of the reduction in early AIDS-related deaths, HIV has become a chronic disease manifesting the common components of chronic disease such as inflammation (2, 3), vascular dysfunction (4-6), and oxidative stress (7, 8). The combination of these adverse factors put HIV patients at increased risk of myocardial infarction (9) compared with age-matched subjects over the long term. A portion of this adverse cardiovascular risk has been linked to therapies necessary to survival in HIV. Several studies suggest that early protease inhibitors might increase the risk of myocardial infarction (10). The leading theory linking early PIs to myocardial infarction stemmed from the relationship between protease inhibitor use and the onset of an atherogenic dysmetabolism including the development of insulin resistance, dyslipidemia, and oxidative stress (11-14). With the advent of newer PIs, and the avoidance of certain NRTIs, the issue of lipodystrophy and insulin resistance has largely been resolved (15).

In contrast to the older protease inhibitors, atazanavir induces neither insulin resistance (16) or dyslipidemia (17). In addition, atazanavir has a property unique among protease inhibitors: elevation of unconjugated bilirubin by inhibiting the enzyme uridine diphosphate glucuronyltransferase (UGT) 1A1 (18). Bilirubin, thought to be solely a waste product, is a potent intracellular antioxidant and may provide a cardiovascular protective effect (19, 20). We have demonstrated that higher levels of bilirubin within the normal range are associated with reduced rates of stroke and peripheral artery disease (21, 22). Patients with Gilbert’s Syndrome (chronic elevations of bilirubin as a result of genetically reduced UGT 1A1) have a lower rate of myocardial infarction compared with age-matched controls (23).

The benefit of atazanavir may be particularly important with the aging of the HIV population. More than 1/4^th^ of all patients with HIV are >50 years of age (24) and the sustained use of ART suggests that the aging of this population will continue, with 50% of the HIV population being older than 50 by 2015. Aging, per se, is an important cardiovascular risk factor and is associated with higher levels of oxidative stress (25-27) and endothelial dysfunction (28). It is plausible that use of atazanavir may improve vascular function through reductions in oxidative stress, ultimately reducing the rate of cardiovascular complications in patients with HIV. Accordingly, we hypothesize that the use of atazanavir in stable HIV patients over the age of 45 years will improve endothelial dysfunction and reduce oxidative stress compared with continuing the current therapy.

**Hypothesis**

We hypothesize that older subjects with HIV randomly assigned to atazanavir will have increased bilirubin levels, reduced oxidative stress, and improved flow-mediated, endothelium-dependent vasodilation compared to subjects not switched to atazanavir.

**Primary Objective**

The primary goal of this study is to determine whether switching to atazanavir will improve vascular endothelial function in older subjects (>45 years) compared to continuing nonatazanavir based therapy.

**Secondary Objectives**

The secondary objective of this study is to determine whether switching to atazanavir reduces oxidative stress, inflammation, and insulin resistance compared with nonatazanavir ART.

In a second analysis, we will recruit 30 stable subjects on atazanavir for more than 6 months meeting the primary objective entry criteria, to perform a single vascular function and metabolic evaluation to determine if chronic atazanavir, when compared to nonatazanavir regimens has lower levels of oxidative stress and improved vascular endothelial function.

**Treatment**

On entry to the study, patients will be randomized to continue their current “key third drug” component of ART or to substitute atazanavir 300 mg daily with ritonavir 100 mg daily (to be taken with food) for 28 days. All patients will continue to receive ongoing co-formulated tenofovir/emtricitabine.

**Trial Population**

Clinically-stable, HIV-infected men and women, 45 years of age or older, who are receiving suppressive ART consisting of co-formulated tenofovir/emtricitabine plus a nonatazanavir/ ritonavir third agent.

For the second protocol, HIV-infected men and women, 45 year of age or older, who are receiving suppressive ART consisting of co-formulated tenofovir/emtricitabine plus atazanavir/ritonavir.

**Sample Size**

It is estimated that the substitution of atazanavir and increase in bilirubin will increase endothelium-dependent, flow-mediated vasodilation FMD by 3-4%. With an estimated standard deviation of the measurement of 4% and a 1:1 randomization scheme, this yields a power of 81-96% with a sample size of 60 subjects in a parallel design trial.

For aim 2, we expect a similar difference in FMD to be noted between the 30 subjects on atazanavir and the 60 subjects at study entry for the primary aim, thus making the comparison 1:2, yielding a power range of 87-99% to detect a difference in flow-mediated vasodilation.

**Key Inclusion Criteria**

- Age ≥ 45 years
- Stable non-atazanavir-containing regimen consisting of co-formulated tenofovir/emtricitabine as the NRTIs plus a third agent for 3 months or longer. The third agent can be any FDA-approved PI, NNRTI, or raltegravir.
- HIV RNA < 50 cop/mL at screening and at least once within the prior year,
- No treatment interruptions > 7 days in the 3 months prior to study entry

**Key Exclusion Criteria**

- Prior treatment failure on or intolerance to atazanavir
- Known or suspected resistance to atazanavir
- Patients with Gilbert’s Syndrome or elevated bilirubin levels (> 1.5 mg/dL) at baseline (for the randomized trial)
- Receiving ART different from co-formulated tenofovir/emtricitabine plus third agent (PI, NNRTI, or raltegravir) regimen
- Current receipt of proton-pump inhibitor therapy
- Recent initiation of hormones or immunomodulators

**Study Assessments**

Evaluation of vascular function in conduit vessels: Vascular function of the brachial artery will be measured in all subjects as reported previously by the applicants and as recommended by a recent task force (29, 30). To assess endothelium-dependent vasodilation, measurements of brachial artery diameter will be made under basal conditions and during reactive hyperemia following five minutes of an ischemic stimulus applied by sphygmomanometric cuff. Reactive hyperemia increases brachial artery blood flow. Flow is a physiologic stimulus that releases nitric oxide from the endothelium. Forearm ischemia will be induced by inflating a blood pressure cuff to suprasystolic pressures for 5 minutes. This results in vasodilation of the downstream resistance vessels. Following release of the cuff, there is a 5 to 8-fold increase in flow through the brachial artery, i.e., reactive hyperemia. We have found that the maximal increase in brachial artery diameter occurs at one minute of reactive hyperemia and that this dilation is mediated by nitric oxide (31). Endothelium-independent vasodilation will be assessed by measuring brachial artery diameter under basal conditions and 3 minutes following the administration of sublingual nitroglycerin (0.4 mg) (32). Maximal brachial artery dilation occurs 3 to 4 minutes after the administration of sublingual nitroglycerin. We have found that this technique yields an interobserver variability of 0.05±0.16% and intraobserver variability of 0±0.15%.

**Correlative Studies**

We will measure blood-based markers of oxidative stress (myeloperoxidase, malondialdehyde, and bilirubin), inflammation (high sensitivity C-reactive protein and tumor necrosis factor alpha receptor 2), and insulin resistance (fasting insulin and glucose). Each of these tests will be correlated with baseline and change in vascular function.

**Data and Statistical Plan**


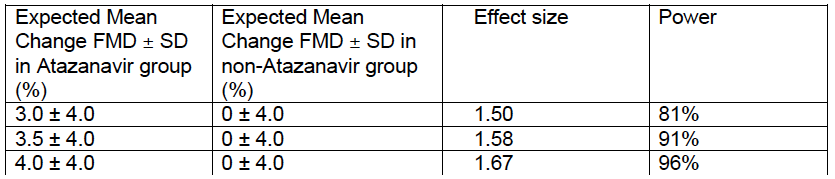
The primary endpoint of this study will be endothelium-dependent, flow-mediated vasodilation of the brachial conduit artery in subjects randomized to atazanavir compared with subjects continuing their current therapy. Nitroglycerin-mediated, endothelium independent vasodilation will be a secondary endpoint. The analysis begins with descriptive and graphical summaries of each outcome measure at pre-treatment, treatment at 7 days, and post treatment and difference from pre to post treatment. The assessment of the normality of each outcome will be performed by plotting the raw data and transformation will be applied if necessary. The primary analysis of between-subject effects will be assessed using student t tests. Sample size calculation: This protocol will be powered to detect a statistically significant difference in both resistance arteriole and conduit artery studies. We will enroll 60 subjects for the randomized portion of this protocol. Thirty subjects will receive atazanavir and 30 will continue to receive their baseline ART. Nitric oxide accounts for 50-60% of the increase in vasodilation to reactive hyperemia (31). It is anticipated that atazanavir will improve absolute endothelium-dependent vasodilation approximately 3% during flow mediated vasodilation. (Please see the table to see a range of power based on effect.)

Analysis of markers of bilirubin, oxidant stress, inflammation, and insulin sensitivity: As key secondary endpoints, the analysis begins with descriptive and graphical summaries of each outcome measure at pre treatment, post treatment and difference from pre to post treatment. The assessment of the normality of each outcome will be performed by plotting the raw data and transformation will be applied if necessary. The primary analysis of between-subject effect will be assessed using paired student t tests. A Bonferroni correction will be applied to account for multiple comparisons. The relationship between changes in secondary markers and changes in vascular function will be assessed using multivariate analysis of variance (MANOVA).

For the second protocol, vascular function for the 30 subjects stable on atazanavir will be compared to the baseline vascular function of the 60 subjects enrolled for the randomized protocol. The analysis plan, including descriptive and graphical summaries, testing of normality with transformation as needed, and between subject t testing will be applied similarly as in randomized trial.

**References**

1. Porter K, Babiker A, Bhaskaran K, Darbyshire J, Pezzotti P, Porter K, et al. Determinants of survival following HIV-1 seroconversion after the introduction of HAART. Lancet. 2003 Oct 18;362(9392):1267-74.

2. Lebech AM, Kristoffersen US, Wiinberg N, Kofoed K, Andersen O, Hesse B, et al. Coronary and peripheral endothelial function in HIV patients studied with positron emission tomography and flow-mediated dilation: relation to hypercholesterolemia. Eur J Nucl Med Mol Imaging. 2008 Nov;35(11):2049-58.

3. Miller TL, Somarriba G, Orav EJ, Mendez AJ, Neri D, Schaefer N, et al. Biomarkers of vascular dysfunction in children infected with human immunodeficiency virus-1. J Acquir Immune Defic Syndr. 2010 Oct 1;55(2):182-8.

4. Blanco JJ, Garcia IS, Cerezo JG, de Rivera JM, Anaya PM, Raya PG, et al. Endothelial function in HIV-infected patients with low or mild cardiovascular risk. J Antimicrob Chemother. 2006 Jul;58(1):133-9.

5. Torriani FJ, Komarow L, Parker RA, Cotter BR, Currier JS, Dube MP, et al. Endothelial function in human immunodeficiency virus-infected antiretroviral-naive subjects before and after starting potent antiretroviral therapy: The ACTG (AIDS Clinical Trials Group) Study 5152s. J Am Coll Cardiol. 2008 Aug 12;52(7):569-76.

6. Stein JH, Merwood MA, Bellehumeur JL, Aeschlimann SE, Korcarz CE, Underbakke GL, et al. Effects of pravastatin on lipoproteins and endothelial function in patients receiving human immunodeficiency virus protease inhibitors. Am Heart J. 2004 Apr;147(4):E18.

7. Ross AC, Rizk N, O'Riordan MA, Dogra V, El-Bejjani D, Storer N, et al. Relationship between inflammatory markers, endothelial activation markers, and carotid intima-media thickness in HIV-infected patients receiving antiretroviral therapy. Clin Infect Dis. 2009 Oct 1;49(7):1119-27.

8. Suresh DR, Annam V, Pratibha K, Prasad BV. Total antioxidant capacity--a novel early bio-chemical marker of oxidative stress in HIV infected individuals. J Biomed Sci. 2009;16:61.

9. Friis-Moller N, Sabin CA, Weber R, d'Arminio Monforte A, El-Sadr WM, Reiss P, et al. Combination antiretroviral therapy and the risk of myocardial infarction. N Engl J Med. 2003 Nov 20;349(21):1993-2003.

10. Lang S, Mary-Krause M, Cotte L, Gilquin J, Partisani M, Simon A, et al. Impact of individual antiretroviral drugs on the risk of myocardial infarction in human immunodeficiency virus-infected patients: a case-control study nested within the French Hospital Database on HIV ANRS cohort CO4. Arch Intern Med. 2010 Jul 26;170(14):1228- 38.

11. Hadigan C, Borgonha S, Rabe J, Young V, Grinspoon S. Increased rates of lipolysis among human immunodeficiency virus-infected men receiving highly active antiretroviral therapy. Metabolism. 2002 Sep;51(9):1143-7.

12. Petit JM, Duong M, Duvillard L, Florentin E, Portier H, Lizard G, et al. LDL-receptors expression in HIV-infected patients: relations to antiretroviral therapy, hormonal status, and presence of lipodystrophy. Eur J Clin Invest. 2002 May;32(5):354-9.

13. Noor MA, Seneviratne T, Aweeka FT, Lo JC, Schwarz JM, Mulligan K, et al. Indinavir acutely inhibits insulin-stimulated glucose disposal in humans: a randomized, placebo-controlled study. AIDS. 2002 Mar 29;16(5):F1-8.

14. Hurwitz BE, Klimas NG, Llabre MM, Maher KJ, Skyler JS, Bilsker MS, et al. HIV, metabolic syndrome X, inflammation, oxidative stress, and coronary heart disease risk : role of protease inhibitor exposure. Cardiovasc Toxicol. 2004;4(3):303-16.

15. Guffanti M, Caumo A, Galli L, Bigoloni A, Galli A, Dagba G, et al. Switching to unboosted atazanavir improves glucose tolerance in highly pretreated HIV-1 infected subjects. Eur J Endocrinol. 2007 Apr;156(4):503-9.

16. Dube MP, Shen C, Greenwald M, Mather KJ. No impairment of endothelial function or insulin sensitivity with 4 weeks of the HIV protease inhibitors atazanavir or lopinavirritonavir in healthy subjects without HIV infection: a placebo-controlled trial. Clin Infect Dis. 2008 Aug 15;47(4):567-74.

17. Murphy RL, Berzins B, Zala C, Fichtenbaum C, Dube MP, Guaraldi G, et al. Change to atazanavir/ritonavir treatment improves lipids but not endothelial function in patients on stable antiretroviral therapy. AIDS. 2010 Mar 27;24(6):885-90.

18. Dekker D, Dorresteijn MJ, Pijnenburg M, Heemskerk S, Rasing-Hoogveld A, Burger DM, et al. The Bilirubin-Increasing Drug Atazanavir Improves Endothelial Function in Patients With Type 2 Diabetes Mellitus. Arterioscler Thromb Vasc Biol. 2010 Nov 18.

19. Stocker R, Yamamoto Y, McDonagh AF, Glazer AN, Ames BN. Bilirubin is an antioxidant of possible physiological importance. Science. 1987 Feb 27;235(4792):1043-6.

20. Sedlak TW, Saleh M, Higginson DS, Paul BD, Juluri KR, Snyder SH. Bilirubin and glutathione have complementary antioxidant and cytoprotective roles. Proc Natl Acad Sci U S A. 2009 Mar 31;106(13):5171-6.

21. Perlstein TS, Pande RL, Beckman JA, Creager MA. Serum total bilirubin level and prevalent lower-extremity peripheral arterial disease: National Health and Nutrition Examination Survey (NHANES) 1999 to 2004. Arterioscler Thromb Vasc Biol. 2008 Jan;28(1):166-72.

22. Perlstein TS, Pande RL, Creager MA, Weuve J, Beckman JA. Serum total bilirubin level, prevalent stroke, and stroke outcomes: NHANES 1999-2004. Am J Med. 2008 Sep;121(9):781-8 e1.

23. Vitek L, Jirsa M, Brodanova M, Kalab M, Marecek Z, Danzig V, et al. Gilbert syndrome and ischemic heart disease: a protective effect of elevated bilirubin levels. Atherosclerosis. 2002 Feb;160(2):449-56.

24. Trends. Aging HIV population faces unique health challenges, officials say. AIDS Policy Law. 2010 Oct;25(11):1, 4.

25. Andriollo-Sanchez M, Hininger-Favier I, Meunier N, Venneria E, O'Connor JM, Maiani G, et al. Age-related oxidative stress and antioxidant parameters in middle-aged and older European subjects: the ZENITH study. Eur J Clin Nutr. 2005 Nov;59 Suppl 2:S58-62.

26. Miwa M, Matsumaru H, Akimoto Y, Naito S, Ochi H. Quantitative determination of urinary 8-hydroxy-2'-deoxyguanosine level in healthy Japanese volunteers. Biofactors. 2004;22(1-4):249-53.

27. Mutlu-Turkoglu U, Ilhan E, Oztezcan S, Kuru A, Aykac-Toker G, Uysal M. Agerelated increases in plasma malondialdehyde and protein carbonyl levels and lymphocyte DNA damage in elderly subjects. Clin Biochem. 2003 Jul;36(5):397-400.

28. Gerhard M, Roddy MA, Creager SJ, Creager MA. Aging progressively impairs endothelium-dependent vasodilation in forearm resistance vessels of humans. Hypertension. 1996 Apr;27(4):849-53.

29. Beckman JA, Liao JK, Hurley S, Garrett LA, Chui D, Mitra D, et al. Atorvastatin restores endothelial function in normocholesterolemic smokers independent of changes in low-density lipoprotein. Circ Res. 2004 Jul 23;95(2):217-23.

30. Corretti MC, Anderson TJ, Benjamin EJ, Celermajer D, Charbonneau F, Creager MA, et al. Guidelines for the ultrasound assessment of endothelial-dependent flow mediated vasodilation of the brachial artery: a report of the International Brachial Artery Reactivity Task Force. J Am Coll Cardiol. 2002 Jan 16;39(2):257-65.

31. Owens CD, Wake N, Conte MS, Gerhard-Herman M, Beckman JA. In vivo human lower extremity saphenous vein bypass grafts manifest flow mediated vasodilation. J Vasc Surg. 2009 Nov;50(5):1063-70.

32. Corretti MC, Anderson TJ, Benjamin EJ, Celermajer D, Charbonneau F, Creager MA, et al. Guidelines for the ultrasound assessment of endothelial-dependent flowmediated vasodilation of the brachial artery: a report of the International Brachial Artery Reactivity Task Force. J Am Coll Cardiol. 2002 Jan 16;39(2):257-65.
